# Complete mitochondrial genome and the phylogenetic position of the *Sphaeroma* sp. (Crustacea, Isopod, Sphaeromatidae)

**DOI:** 10.1080/23802359.2019.1687350

**Published:** 2019-11-08

**Authors:** Mingliu Yang, Tingwei Gao, Ding Hui, Xiao Chen, Wenai Liu

**Affiliations:** a Guangxi Academy of Sciences, Guangxi Mangrove Research Center, Guangxi Key Lab of Mangrove Conservation and Utilization, Beihai, PR China;; b College of Marine Sciences, South China Agricultural University, Guangzhou, PR China

**Keywords:** *Sphaeroma* sp, mitochondrial genome, Sphaeromatidae

## Abstract

The complete mitogenome of *Sphaeroma* sp. (Crustacea, Isopod, Sphaeromatidae) was determined in this study. The total length of mitogenome was 15,839 bp, and contained 13 protein-coding genes, 21 tRNA genes, 2 rRNA genes, 1 control region, and 2 unknown fragments which longer than 200 bp. The nucleotide composition was 25.80% A, 16.56% C, 25.94% G and 31.67% T. Six initiation codons (ATA, ATC, ATG, ATT, ACG, GTG) and three termination codons (TAA, TAG, TA–) were used in the protein-coding genes. The length of tRNA genes ranged from 52 to 65 bp, and tRNA-*Arg* was not identified. The phylogenetic result showed *Sphaeroma* sp. was closely related to *Sphaeroma terebrans* with high bootstrap value supported.

Sphaeromatidae is one of the largest families in Isopod (979 species in 125 genera) (WoRMS Editorial Board 2019). A part of Sphaeromatidae genetic data had been sequenced, nonetheless, only one complete mitochodrial genome was published in this family, which was *Sphaeroma terebrans* collected from China (Yang et al. 2019). Here, we determined the complete mitogenome of *Sphaeroma* sp. and analyzed the phylogenetic position of this species, hoping that could contribute to the molecular and phylogenetic study about isopod crustaceans.

Specimens of *Sphaeroma* sp. were collected from Beihai Coastal National Wetland Park, Beihai, Guangxi Province, China (21°24′41.11″N, 109°10′23.26″E) on October 2016 and preserved in 95% ethanol. After morphological identification, the specimen was deposited in Guangxi Mangrove Research Center with a voucher number DG20161009. The experimental protocol, data analysis methods and Maximum likelihood (ML) phylogenetic reconstruction followed Yang et al. (2019). The outgroup *Metacrangonyx longipes* and 12 species of Isopod mitogenomes available in GenBank were used for the phylogenetic reconstruction.

The complete mitogenome of *sphaeroma* sp. was 15, 839 bp in length (GenBank accession number MN263919) and its overall base composition was 25.80% A, 16.56% C, 25.94% G, and 31.67% T. The mitogenomic variation between *Sphaeroma* sp. and *S. terebrans* (MK460228) was 28.3%. It consisted of 13 protein-coding genes, 21 transfer RNA genes, two rRNA genes, one control region and two unknown fragments which longer than 200 bp. Despite extensive efforts to find secondary structures in control region and unknown fragments, the tRNA-*Arg* gene was not identified in the mitogenome. Except for *Cytb*, *ND1*, *ND4*, and *ND4L* were encoded on the L-strand, the remaining 9 protein-coding genes were encoded on the H-strand. This arrangement of protein-coding genes was similar to *Ligia oceanica* (Kilpert and Podsiadlowski 2006) and *S. terebrans* (Yang et al. 2019). It was important to note that three protein-coding genes started with ATG codon (*ND4*, *ATP6*, and *COX3*), four with ATT codon (*ND4L*, *ND6*, *ND2*, and *ATP8*), two with ATA codon (*ND1* and *ND5*), two with ATC codon (*COX2* and *Cytb*), one with GTG codon (*ND3*), and one with ACG codon (*COX1*). All the protein-coding genes used TAA, TAG, and TA– as termination codons. The 21 tRNA genes interspersed between the rRNAs and protein-coding genes, ranging from 52 to 65 bp. The control region was 813 bp, presenting a high A + T content (62.12%).

Five suborders of the Isopod were showed in the ML tree and most nodes were well supported (Figure 1). The suborder Phreatoicidea was the basal clade. The Flabellifera was sister to the Valvifera. Within the family Sphaeromatidae of the suborder Flabellifera, *Sphaeroma* sp. clustered to *S. terebrans*, and then *S. serratum* clustered to this clade. *Ligia oceanica* clustered to the clades of Valvifera and Flabellifera instead of Oniscidea, it suggested that the suborder Oniscidea was a polyphyletic group. In conclusion, the complete mitogenome of *Sphaeroma* sp. could provide essential and important DNA molecular data for further studies of Isopod phylogeny.

**Figure 1.**
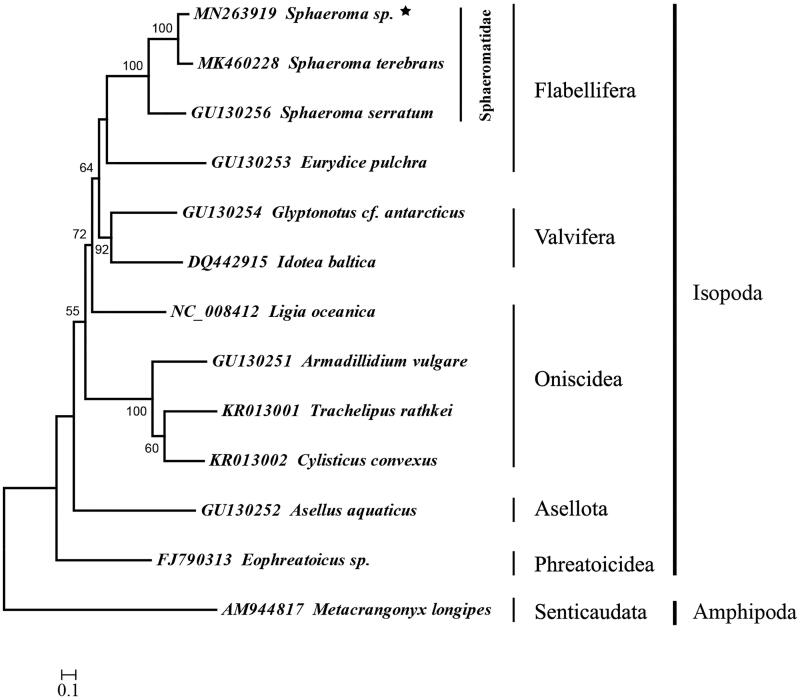
Phylogenetic position of *Sphaeroma sp*. (Asterisked). *Metacrangonyx longipes* was selected as the out group. The mitochondrial genome of the selected species for tree construction were retrieved from the GenBank, and the gene’s accession number was listed before the species name.
